# Stages of use: consideration, initiation, utilization, and outcomes of an internet-mediated intervention

**DOI:** 10.1186/1472-6947-10-73

**Published:** 2010-11-23

**Authors:** Teresa ML Chiu, Gunther Eysenbach

**Affiliations:** 1 Department of Rehabilitation Sciences, Hong Kong Polytechnic University, Hung Hom, Hong Kong, PR China; 2 Centre for Global eHealth Innovation, Toronto, R. Fraser Elliott Building, 4th Floor 190 Elizabeth Street Toronto, Ontario, Canada; 3 Department of Health Policy, Management, and Evaluation, University of Toronto, 250 College Street, Toronto, Ontario, Canada

## Abstract

**Background:**

Attrition, or nonuse of the intervention, is a significant problem in e-health. However, the reasons for this phenomenon are poorly understood. Building on Eysenbach's "Law of Attrition", this study aimed to explore the usage behavior of users of e-health services. We used two theoretical models, Andersen's Behavioral Model of Health Service Utilization and Venkatesh's Unified Theory of Acceptance and Use of Technology, to explore the factors associated with uptake and use of an internet-mediated intervention for caregivers taking care of a family member with dementia.

**Methods:**

A multiphase, longitudinal design was used to follow a convenience sample of 46 family caregivers who received an e-health intervention. Applying the two theories, usage behavior was conceptualized to form four stages: consideration, initiation, utilization (attrition or continuation), and outcome. The variables and measurement scales were selected based on these theories to measure the sociodemographic context, technology aptitudes, and clinical needs of the caregivers.

**Results:**

In the *Consideration Stage*, caregivers who felt that the information communication technology (ICT)-mediated service was easy to use were more likely to consider participating in the study (*p *= 0.04). In the *Initiation Stage*, caregivers who showed greater technology acceptance were more likely to initiate service earlier (*p *= 0.02). In the *Utilization Stage*, the frequent users were those who had a more positive attitude toward technology (*p *= 0.04) and a lower perceived caregiver competence (*p *= 0.04) compared with nonusers. In the *Outcome Stage*, frequent users experienced a decline in perceived burden compared with an escalation of perceived burden by nonusers (*p *= 0.02).

**Conclusions:**

We illustrate a methodological framework describing how to develop and expand a theory on attrition. The proposed framework highlighted the importance of conceptualizing e-health "use" and "adoption" as dynamic, continuous, longitudinal processes occurring in different stages, influenced by different factors to predict advancement to the next stage. Although usage behavior was influenced mainly by technological factors in the initial stages, both clinical and technological factors were equally important in the later stages. Frequency of use was associated with positive clinical outcomes. A plausible explanation was that intervention benefits motivated the caregivers to continue the service and regular use led to more positive clinical outcome.

## Background

Information communication technologies (ICT) have been used to provide a broad range of e-health services, including providing supportive interventions to people with chronic diseases and their family caregivers. A prerequisite for successful e-health services (and ultimately changes in the outcomes) is that the service is actually being used. However, high attrition rates from e-health trials, which may be a natural or typical feature of e-health services, have been reported in ICT-mediated interventions. Eysenbach [1], in his influential "Law of Attrition" (LOA) paper, called for a "science of attrition" and categorized attrition into *nonusage attrition *and *dropout attrition*; *nonusage attrition *refers to the discontinuation of services, and *dropout attrition *refers to the loss of follow-up data in the context of a trial.

This paper focuses on the exploration of attrition. A number of other terms have been used for identical or related concepts, including "adherence", "compliance", "dosage", "usage behavior", "adoption", "uptake", "engagement", "retention", and "exposure" [2-11]. Various concrete factors have been mentioned by Eysenbach that may influence attrition, including appropriateness of information, ease of enrolment, ease of drop out, usability issues, "push" factors (e.g., reminders), positive feedback, observable advantages, paid intervention and other incentives, time commitment, competing interventions, external events competing for the participants' attention, peer communications/peer pressure, human contact, and experience of use [1]. Many of these suggested factors broadly fit into existing theories of health service utilization and technology use and adoption, although these conceptual links have not previously been explicated. Empirical findings are required to substantiate these proposed factors, and relationships to existing theories have to be identified to facilitate the development of a more comprehensive framework and theory of attrition, with the ultimate aim being to inform the development and evaluation of attractive, "sticky" and engaging e-health applications that generate minimal attrition and maximum "adherence".

### Empirical findings of ICT-mediated interventions for dementia caregivers

This study uses data from an electronic support intervention for caregivers of dementia patients. In a specific systematic review on networked technologies supporting caregivers of people with dementia, five empirical studies of ICT-mediated interventions for dementia caregivers were identified [12]. Usage behavior was found to vary across studies and was not consistently reported in all studies. In the ComputerLink study, the analysis showed that younger caregivers were more likely to use ComputerLink than older caregivers [13]. Caregivers who had a greater baseline stress were likely to experience a greater decline in stress upon completion of the study [14]. With respect to the Computer Telephone Integration System (CTIS) study, caregivers who dropped out were more depressed at baseline than those who completed the study [15]. In the Telephone Linked Care (TLC) study, the results suggested that the adopters were older male caregivers, better educated, and more proficient in using TLC than nonadopters [16]. In the AlzOnline study, 8% of the participants dropped out shortly after the screening interview, and another 15% discontinued within three or fewer classes [17]. The primary reasons for this attrition were competing work-related responsibilities and heavy caregiving responsibilities. In the Caring for Others study, 29% of the participants were lost to follow-up [18]. The empirical findings from these studies provided limited explanations of the relationship between attrition and other e-health factors. Therefore, we identified two theoretical models to conceptualize the attrition phenomenon in e-health service use.

### Andersen's Behavioral Model of Health Service Utilization (BMHSU)

Andersen's Behavioral Model of Health Service Utilization (BMHSU) [19,20] is the most frequently used theoretical model for predicting and explaining health services use. The model consists of three determinant factors: *predisposing, enabling*, and *needs factors. Predisposing factors *are exogenous factors such as demographics, social structure, and health beliefs. *Enabling factors *are necessary but not sufficient conditions for service use. They include community and personal enabling resources. *Needs factors *must be present for service use to happen. There are two types of needs variables: *evaluated needs *and *perceived needs*. The needs experienced by the caregivers are the perceived needs.

### Venkatesh's Unified Theory of Acceptance and Use of Technology (UTAUT)

Venkatesh's Unified Theory of Acceptance and Use of Technology (UTAUT) explains the intention to adopt or use information technology [21]. There are four core constructs: (1) *Performance expectancy*, which is a person's belief that a new technology will improve task performance. This is the main predictor of technology acceptance. (2) *Effort expectancy*, which is the degree of ease associated with the use of the new technology. (3) *Social influence*, which measures the degree to which someone important to a person influences his or her decision to use the new technology. (4) *Facilitating conditions*, which is the support available to facilitate a person's use of the new technology. These four constructs affect *intention to use*, which in turn predicts technology usage. Performance expectancy and effort expectancy explain a greater proportion of the variance than the other factors [21]. These ideas can be applied to the context of this study: if a caregiver perceives that the new e-health service is easy to use and is useful, the caregiver is more likely to use the service compared with other caregivers who feel that the service is difficult to use or not useful.

### Research aims and objectives

This paper explores the predictors of usage behavior for an e-health service. For the purpose of this paper, we conceptualized different "stages of use" (consideration to use, intention to use, utilization with attrition or continuation, and outcome stage; see description under "Methods"), realizing that in each stage, different factors may play different roles and become less or more important to predict advancement to the next stage. Four research objectives were identified that addressed the usage behavior in the different proposed stages of use:

1. To explore factors associated with a caregiver's decision to use the online support (intention to use).

2. To explore factors associated with the time taken to initiate the service (initiation)

3. To explore factors associated with service utilization (nonusage attrition and frequency of use)

4. To explore the usage behavior associated with outcomes.

The current study is intended to be an explorative study. It was not feasible to measure all possible, theory-driven factors in a single study because the questionnaires for the participants would be too long. We realize that this study will not identify a comprehensive list of factors; rather, the findings of this study need to be complemented by other studies, for which this paper may provide a methodological framework.

## Methods

### Design

**Figure 1:**
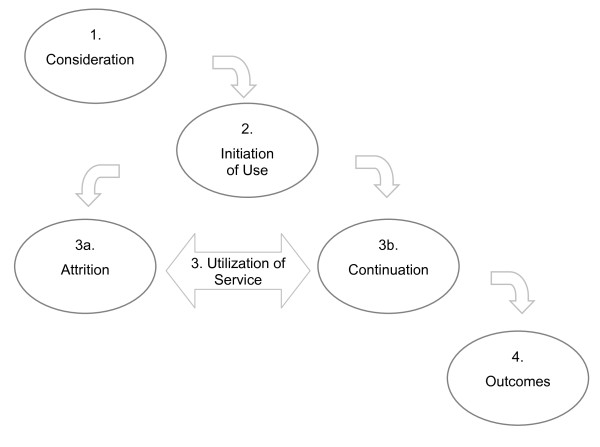
Stages of use

We used a multiphase, longitudinal study design. The four objectives were addressed in the four proposed stages of use (Figure 1). Applying Venkatesh's UTAUT and Anderson's BMHSU, we conceptualized that caregivers will go through the stages of consideration, initiation, utilization, and outcomes.

### Conceptualization of the four stages of use

In the *consideration stage*, interested caregivers evaluate the service first before deciding whether they will use the service or not. The decision is based on information available about the intervention/service. In the context of a trial, this decision would be influenced by information provided in an informed consent document. In the context of a commercial product, caregivers may base the decision on information in an advertisement. Peer pressure and social norms (expectations from others, including health professionals) may also play an important role in this stage. Caregivers who decide to use the service will act upon the decision. For example, they will sign an informed consent form, purchase a service, or order/create a user account.

The second stage is the *initiation stage*, in which the caregivers start to use the service. To measure this usage behavior, one can for example measure how quickly a user begins using a newly introduced or purchased service (e.g., first login or first meaningful action on an e-health service) after the initial agreement to use it. It is unclear what factors make users who have originally agreed to participate (or who purchased a service) actually use the service or change their mind, but factors that likely play a role here are the emergence of new information (influencing performance expectancy), competing interests (lack of time), change of perceived or actual needs, lack of peer pressure originally present in the consideration stage, or simply forgetting about it.

Following the initiation of service, the user begins to actively use the service. This is the *utilization *stage. Venkatesh's UTAUT suggests that the acceptance of a technology affects ongoing usage behavior. That is, the experience of using a technology now directly influences the perception of the ease of use and usefulness of the technology, which consequently affects the intention to continue using it. Andersen's BMHSU also describes how a change of perceived needs can directly influence whether a person continues to need the service. An indirect impact after use is the change of the belief in the health service (predisposing factor), which affects the frequency of service utilization. Hence, the third stage is conceptualized as the *utilization stage *(Figure 1), in which the users actively engage in the intervention. Within this stage, the users may choose from one of two paths, *attrition *or *continuation*. If the users discontinue the service, the intervention is ended before completion. If the users continue to engage in the intervention until their needs are met, they eventually reach the final stage.

Finally, how usage behavior influences clinical outcomes is an integral part of the concept. As stated in Venkatesh's UTAUT, the ultimate goal of improving technology acceptance is to improve a person's work performance, while the aim of improving service utilization in Andersen's BMHSU is to improve a person's clinical outcomes. Conversely, being able to experience and observe changes in clinical or psychological outcomes provides a powerful motivation for individuals to continue use (or to stop use, if they feel a plateau has been reached or the intervention is no longer required to maintain the changes). Because attaining a positive outcome is the ultimate goal of e-health interventions, the fourth and final stage of use is the *outcome stage *(Figure 1).

### Intervention

The intervention program consisted of an exchange of e-mails between Chinese family caregivers (informal caregivers, such as a daughter, a son, or a spouse) of elderly patients with Alzheimer's disease and related dementia and Chinese professional clinicians (occupational therapists) for a period of 6 months. The intervention was offered by two online therapists who had more than two years of relevant clinical experience and received regular clinical supervision during the study. E-mail contact was the only form of communication between the therapists and caregivers throughout the program. The study design, a description of the intervention program, and the clinical results have been reported elsewhere [22]. The study received ethics approval from the University of Toronto, COTA Health, and the Yee Hong Center for Geriatric Care (Yee Hong) in Toronto, Canada.

### Participants

**Figure 2:**
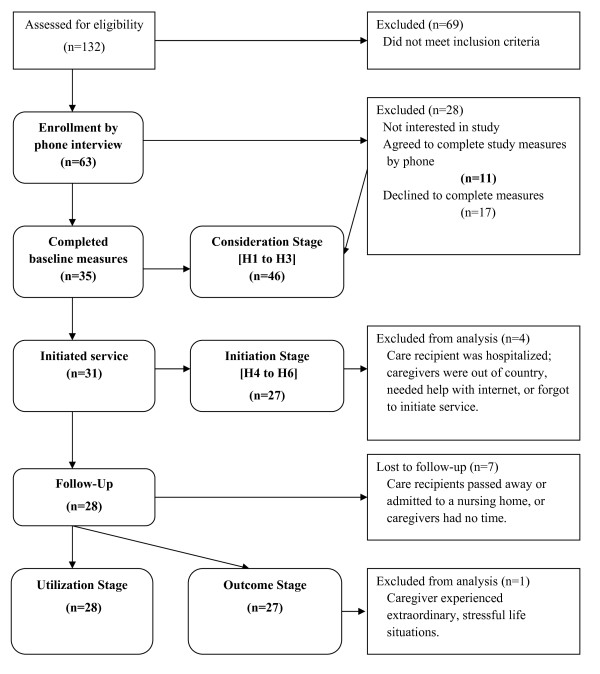
Sample selection and data collection flow diagram

Chinese family caregivers were recruited from Yee Hong's adult day programs, a not-for-profit community organization in the Greater Toronto Area using a convenience sampling strategy. The sample consisted of unpaid, informal Chinese caregivers who took care of a family member suffering from dementia who lived at home (i.e., not in a nursing home). To be eligible, family caregivers had to meet the following criteria: (a) be 18 years or older, (b) be able to speak, read and/or write Chinese, (c) be caring for a family member with dementia living in the community, (i.e., not an institutional setting), and (d) have Internet access. Figure 2 shows the sample selection and data collection procedures in each stage. The sample size was estimated to be 29 to test the postintervention change in the Burden Scale for Family Caregivers (BSFC) score in the main study [22]. The sample size calculation was based on an 80% power to detect a change of 7 or more in the BSFC scores (alpha 0.05; standard deviation: 9.5).

### Variables and instruments

Various validated instruments or other measurements were used to collect information on dependent and independent variables for multivariate analyses, with the goal being to identify those independent variables that predicted the dependent variables. The Chinese versions of the instruments were used when available. Otherwise, the instruments were translated into Chinese for this study.

### Dependent variables

Intention to use (Stage 1/Objective 1) was measured as the consent decisions (yes and no) of qualified caregivers. Initiation of use (Stage 2/Objective 2) measured how long it took a caregiver to activate the e-mail account after the user package was sent out (by mail and e-mail). Nonusage attrition and frequency of use (Stage 3/Objective 3) measured the number of e-mails a caregiver sent to the online therapist. The frequency of use was divided into three user groups: nonusers (never sent an e-mail), occasional users (one to two e-mails), and frequent users (three or more e-mails). Outcome of use (Stage 4/Objective 4) was defined as the change in caregiver burden after service use. The BSFC [23], which measures the perceived burden of caregivers who help a family member at home, was used to assess the outcome. A Chinese version of the BSFC developed for this study had a Cronbach's alpha of 0.91. The BSFC was measured at baseline and at 6 months.

### Independent variables

The UTAUT [21] measures technology acceptance and includes four subscales: performance expectancy, effort expectancy, social influence, and facilitating conditions. The Caregiver Competence Measure (CCM) [23] is a one-item measure involving the statement "I feel I have the skills and knowledge to be a competent caregiver." The Older American Resources and Services (OARS) Multidimensional Functional Assessment Questionnaire (OARS) [24] measures the functioning level of the care recipients. The Revised Memory and Behavior Problems Checklist (RMBPC) [25] measures how caregivers react to problem behaviors from care recipients. The Self-Rated Health (SRH) scale [26] measures caregivers' ratings of their own health. The Technology Profile Inventory (TPI) [27] measures a person's attitude toward information technology. All independent variables were measured at baseline after the caregivers consented to participate in the study.

### Data collection

#### Objective 1

Intention to use. Eligible caregivers completed the study measures (BSFC, UTAUT, CCM, OARS, RMBPC, SRH, TPI) and gave their written consent to participate. Caregivers who were eligible but not interested in the study were interviewed in this stage to answer questions about their caregiving situation and why they were not interested. They also completed the study measures.

#### Objective 2

Initiation of use. Each caregiver received a user package in the mail that contained a user name, password, and a user manual. The caregivers were invited to activate their account within two weeks. Caregivers who did not activate their account within two weeks received calls to follow up. The number of days between providing the user package and the first login was recorded as a dependent variable.

#### Objective 3

Nonusage attrition and frequency of use. After activating the e-mail account, the caregivers could e-mail the therapist at a time that was convenient to them using their choice of language. The number of e-mails was recorded as a dependent variable.

#### Objective 4

Outcome of use. The participants received the postintervention questionnaire containing the BSFC in the mail 6 months post-service initiation. They received reminder calls to return the questionnaire. The BSFC change score was used as a dependent variable.

### Data analysis

Chi-square tests, ANOVA (analysis of variance), *t*-tests, and linear regression were used to select variables in the univariate analysis in each stage. Multivariate logistic regression analyses were conducted using the selected variables, and confounding effects were analyzed. An intention-to-treat analysis was conducted in the outcome stage.

## Results

### Study participants (Figure 2)

#### Objective 1

Intention to use. The phone interviewers screened 132 caregivers, and 63 of them (47.7%) qualified to participate. Out of the 63 qualified caregivers, 35 consented and 28 did not. When asked to explain why they were not interested, 19 reported the following reasons: they had no time, they were too stressed, they were too busy, or they knew enough about caregiving already. When the 28 uninterested caregivers were asked to complete the study questionnaire by phone, only 11 of them agreed to do so. The total sample in this stage was 46. Table 1 presents the participant characteristics at baseline.

**Table 1:** The characteristics of participants (N = 46) and uninterested caregivers who completed study measures (N = 21)

Participant characteristics		Consent Groups			
		
		Yes		No*	
		
		N	Col. %	N	Col. %
Gender	F	21	60.0%	4	36.4%
	M	14	40.0%	7	63.6%

Age Group	<50	17	48.6%	6	54.5%
	51-64	14	40.0%	5	45.5%
	65+	4	11.4%	0	0%

Relationship	Daughter	17	48.6%	4	36.4%
	Son	13	37.1%	7	63.6%
	Spouse	2	5.7%	0	0%
	Other	3	8.6%	0	0%

* Caregivers who were not interested in the study but agreed to complete study measures during a phone interview.

#### Objective 2

Initiation of use. Out of the 35 caregivers who gave consent, 31 initiated the services. The four caregivers did not activate the account and another four caregivers who took an unusually long time (i.e., more than 70 days) to activate the account were excluded from the sample. The sample size was 27 in this stage.

#### Objective 3

Service utilization (nonusage attrition and frequency of use). Among the 31 caregivers who initiated the service in Stage 2, 28 completed the postintervention questionnaire and 19 received the intervention. This represented a 3% dropout attrition rate and a 39% nonusage attrition rate.

#### Objective 4

Outcome of use. Twenty-eight participants completed the follow-up measures. Seven participants did not complete the follow-up measures (Figure 2). One completer was excluded from the analysis because of an unusual increase in the BSFC score at the postintervention because of an extraordinary life stress. Therefore, at the postintervention, the sample size was 27.

### Results from the Multivariate Analyses

**Figure 3:**
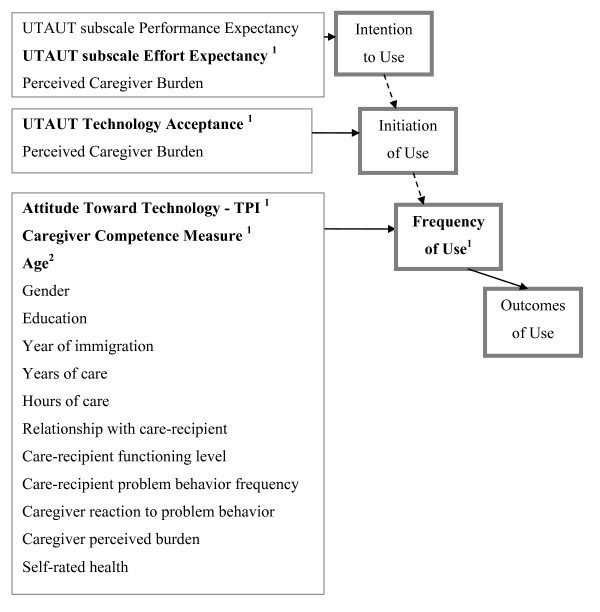
**Factors affecting usage behavior**. ^1 ^Significant in multivariate analysis ^2 ^Significant in univariate analysis but excluded in multivariate analysis

Figure 3 summarizes the factors tested and identified as predictors affecting usage behavior according to the four research objectives and stages.

### Stage 1: Intention to use

In Stage 1, three factors affecting uptake were explored by comparing the consent and nonconsent groups (n = 46). The first factor was the perceived usefulness of the e-mail support service to the caregiver. The mean UTAUT performance expectancy scores of the consent and nonconsent groups were 19.54 and 19.63, respectively, and the difference was not statistically significant. The second factor was the caregivers' perception of the ease of use of the support service. The mean UTAUT effort expectancy score of the consent group was 19.03 and that of the nonconsent group was 21.27, and the difference was statistically significant (*t*-test, *p *= 0.04). The third factor was the caregivers' perceived burden of care. The mean BSFC score of the consent and nonconsent groups were 35.97 and 40.00, and the difference was not statistically significant. A multivariate logistic regression analysis (Table 2) showed the UTAUT performance expectancy and BSFC were not significant (*p *= 0.72 and 0.35, respectively). The only significant factor was the UTAUT effort expectancy (*p *= 0.04), suggesting that caregivers who gave consent to participate gave higher ratings on ease of use.

**Table 2:** A multivariate logistic analysis of the UTAUT subscales and the perceived burden on intention to use (n = 46)

Variables Entered in Logistic Regression Model	Intention to Use (Consent or Not)			
	B	SE	Odds Ratio	95% CI
Constant	7.15	3.71	1276.55	
UTAUT subscale Performance Expectancy (H1)	.05	.14	1.05	.80 to 1.38
UTAUT subscale Effort Expectancy (H2)	-.28	.13	.76	.59 to .98*
Perceived Burden (H3)	-.04	.04	.97	.90 to 1.38

* Caregivers who had higher effort expectancies were less likely to consent (p = 0.04).

### Stage 2: Initiation of use

In this stage, another three factors were examined for their association with how quickly the caregivers began services. The outcome variable was the time taken to log onto the service the first time, and the average time was 12.26 days (*SD *= 8.38; *range *= 1 to 34; *median *= 11.00).

The factors were the caregivers' intention to use (intended duration), the caregivers' acceptance of technology, and the clinical needs of the caregivers. The multivariate linear regression analysis using a backward stepwise procedure showed that both intention to use and clinical needs were not significant (*p *= 0.89 and 0.85, respectively). The only significant variable was the UTAUT technology acceptance score. The results showed that caregivers who had a higher score on the measure started the service earlier than did those with lower scores (*p *= 0.04). Further analysis was conducted to assess whether the following variables might moderate the relationship between technology acceptance and initiation of use: gender, age group, employment, hours of care per week, years of care, age at immigration, language preference, living with care recipient, positive aspects of caregiving, social support, and technology aptitude. The results showed that none of these factors had an effect on the relationship.

### Stage 3: Nonusage attrition and the frequency of use

Objective 3 explored factors associated with service utilization, which was divided into continuation and attrition. In this stage, the participants were divided into three groups based on the frequency of use: nonusers (no e-mail; *n *= 9), occasional users (1 to 2 e-mails; *n *= 8), and frequent users (3 or more e-mails; *N *= 10).

Univariate analysis showed that age group, CCM, and attitude toward technology (TPI) had a statistically significant group effect on usage. Older participants tended to be nonusers, whereas younger participants were more likely to be occasional or frequent users (*p *= 0.047). Nonusers mostly rated that they were competent to give good care, whereas occasional or frequent users felt they were not (*p *= 0.01). When usage frequency increased, the attitude toward technology (TPI) showed an increase in positive attitude (*p *= .012). The difference in TPI scores between nonusers and frequent users was 5.75 (*t *= 3.37, *degree of freedom *[*df *] = 17, *p *= 0.01). However, no group effect was found for the following variables: gender, education, years of immigration, years of care, hours of care, relationship with care recipient, care-recipient functioning level (OARS), problem behavior frequency (RMBPC-Freq), caregiver reaction to problem behaviors (RMBPC-Reaction), caregiver perceived burden (BSFC), self-rated health (SRH), or UTAUT technology acceptance.

The correlations among age group, CCM, and TPI were explored. The results showed that age group and CCM were correlated (Spearman *r = *-4.03; *p *= 0.02), with an older age associated with greater perceived competence. Both older age and greater perceived competence were associated with less frequent use of e-mail. In addition, TPI and age group were found to be correlated (Spearman *r *= -0.40; *p *= 0.04), with an older age associated with a less positive attitude toward technology. Finally, TPI did not have a statistically significant correlation with CCM.

Logistic regression of usage frequency (frequent users vs. nonusers) showed that both CCM (*p *= 0.04) and TPI were associated with usage frequency (*p *= 0.04), but age group was not (*p *= 0.06) (Table 3). Caregivers who were less competent were more likely to be frequent users. The TPI scores were higher among frequent users than among nonusers across both caregiver competence groups.

**Table 3:** Logistic regression model for usage (frequent users vs. nonusers; n = 19)

Variables in the Final Model	B	SE B	Odds Ratio	95% CI
Constant	-20.09	8.88	.024	
Caregiver Competence Measure (CCM)	4.35	2.17	77.79	1.12 to 5415.2*
Attitude toward technology (TPI)	.41	.20	1.51	1.03 to 2.21**

* More competent caregivers were more likely to be nonusers (p = 0.04).

**Caregivers with more positive technology attitude were more likely to be frequent users (p = 0.04).

### Stage 4: Outcome of use

The final stage--the outcome stage--explored the factors associated with clinical outcomes. The mean pre- and postintervention BSFC change score was 1.07 (*t *= -0.63, *df *= 26, *p *= 0.54). Nonusers had an increase in perceived burden at postintervention, whereas occasional users had minimal changes in score and frequent users had a decrease of score. An analysis of variance showed that the differences among the three groups were not statistically significant (*F *= 1.78, *p *= 0.19). However, when the BSFC change scores were compared between nonusers and frequent users (Table 3), the difference was 7.42 and was statistically significant (*t *= 2.50, *df *= 17, *p *= 0.02).

Besides frequency of use, the following variables were tested for their association with the BSFC score: age, gender, employment, education, year of immigration, English proficiency, years of care, hours of care, living together, OARS, SRH, TPI, UTAUT technology acceptance, RMBPC frequency and reaction subscales and CCM (binary). The results showed that none of these factors were statistically significantly associated with the BSFC score.

Intent-to-treat was analyzed by including caregivers who dropped out. Dropout caregivers were assumed to have a BSFC change score of 5.22 (the mean change score of nonusers). After including the seven dropouts, the mean BSFC change score was 2.76, which was not statistically significant (*t *= 1.75, *df *= 34, *p *= 0.09). However, a statistically significant difference between nonusers (*N *= 14) and frequent users (*N *= 10) was found. The difference in BSFC change scores was 7.42 (*t *= 3.15, *df *= 22, *p *= 0.005).

## Discussion and conclusions

This study was based on a study of e-mail support intervention that identified the intervention outcomes and factors associated with the outcomes. Similar to the findings of other e-health studies, the original study showed that e-mail support interventions benefit some but not all caregivers and that these interventions have high nonusage attrition [22]. The results showed that regular engagement in intervention was associated with a reduction in caregiver burden postintervention. Also, traditional beliefs shaped caregivers' needs, and ethno-cultural-linguistic contexts affected system usability and were associated with usage behavior.

This study aimed to explore factors associated with the usage behavior of family caregivers who used an e-health intervention. We conceptualized four stages of use based on the Andersen's BMHSU and Venkatesh's UTAUT.

In the *consideration stage*, caregivers who felt that the service was easy to use were more likely to consider participating in the study. Although the results showed that performance expectancy was not associated with consent decision, this fact does not necessarily mean that the perceived usefulness of e-health service is not important. In fact, a further analysis showed that the UTAUT performance scores of consent and nonconsent groups were 19.54 and 19.63, respectively, indicating that both groups agreed that the service would be useful to them. The findings imply that when caregivers are attracted to a service because of its perceived usefulness, it is the non-user-friendly features that stop them from eventually using it.

In the *initiation stage*, caregivers who had a higher technology acceptance were more likely to begin service earlier. Age has been identified as a factor associated with adoption of e-health service in prior studies [13,16]. However, when both age and attitude toward technology were entered into the regression analysis in this study, age was a weaker predictor compared with attitude toward technology. Measurement of technology acceptance may provide more useful information than age when assessing user profiles.

In the *utilization stage (attrition or continuation)*, caregivers made the decision to continue service based on their clinical needs and technology aptitude. Caregivers with less caregiving competence were more likely to continue service, and those with a more positive attitude toward technology were more likely to continue service. Although only attrition and continuation have been identified in this stage, we have observed other patterns that are more complex and that also influence utilization. In a qualitative study, we interviewed selected caregivers from the original study sample [28] and identified other patterns of use, for example, periods of intensive uses separated by extended nonuse periods. The service utilization pattern could be explained by caregiver's needs, ICT access barriers, and the learning style of the caregivers.

In the *outcome stage*, the results showed that frequent users experienced a decline in perceived burden compared with an escalation of perceived burden by nonusers. Several things may explain this finding. After experiencing the clinical benefits, frequent users were more likely to continue service and eventually benefited more from it. Note that even though the technology and clinical needs factors did not affect the outcomes directly, these factors were associated with the frequency of use. Therefore, motivating users to adhere to an intervention had an indirect impact on the outcomes.

### Limitations

This study was subject to various limitations. As an explorative study, it was not feasible to gather a comprehensive list of all of the possible factors in all of the stages of use. Another limitation was the difficulty faced in collecting data, particularly from noninterested caregivers and from dropouts. While a fairly good recruitment result was achieved by collaborating with a well-respected Chinese community agency and reaching out via phone interviewers, only 11 noninterested caregivers completed the questionnaire, and there were only 9 caregivers in the nonuser study group. The sample size did not have enough power to reach the level of statistical significance required to explore some factors; for example, the clinical needs in the consideration and initiation stages. Despite these limitations, the study has contributed to answering some of the theory-driven questions on usage and attrition behavior supported by empirical findings. It also provides a template for future studies to explore additional factors.

## Conclusion

We proposed a theory-driven and empirically tested "stages of use" framework that explores usage behavior and the associated factors in different phases when family caregivers adopt and use an e-health service. This study contributes to the understanding of not only what factors affect clinical outcomes but also why caregivers use or do not use e-health interventions and what happens in different stages when they use the service. Whereas usage behaviors are influenced mainly by technological factors in the initial stages, both clinical and technological factors are equally important in the later stages. Most importantly, the frequency of use is associated with clinical outcomes. The designers and evaluators of internet-based clinical interventions can use the framework presented here to conceptualize how clinical and technological factors influence the intervention process and clinical outcomes.

## Competing interests

The authors declare that they have no competing interests.

## Authors' contributions

TC designed the study, gathered the data, performed the analysis, interpreted the findings and prepared the initial draft of the manuscript. GE contributed to the study concept, interpretation of findings and revision of manuscript, and supervised TC's research as part of her PhD program. All authors have read and approved the final manuscript.

## Pre-publication history

The pre-publication history for this paper can be accessed here:

http://www.biomedcentral.com/1472-6947/10/73/prepub
